# Strain-Dependent Effect of Macroautophagy on Abnormally Folded Prion Protein Degradation in Infected Neuronal Cells

**DOI:** 10.1371/journal.pone.0137958

**Published:** 2015-09-14

**Authors:** Daisuke Ishibashi, Takujiro Homma, Takehiro Nakagaki, Takayuki Fuse, Kazunori Sano, Hanae Takatsuki, Ryuichiro Atarashi, Noriyuki Nishida

**Affiliations:** Department of Molecular Microbiology and Immunology, Nagasaki University Graduate School of Biomedical Sciences, Nagasaki, Japan; Van Andel Institute, UNITED STATES

## Abstract

Prion diseases are neurodegenerative disorders caused by the accumulation of abnormal prion protein (PrP^Sc^) in the central nervous system. With the aim of elucidating the mechanism underlying the accumulation and degradation of PrP^Sc^, we investigated the role of autophagy in its degradation, using cultured cells stably infected with distinct prion strains. The effects of pharmacological compounds that inhibit or stimulate the cellular signal transduction pathways that mediate autophagy during PrP^Sc^ degradation were evaluated. The accumulation of PrP^Sc^ in cells persistently infected with the prion strain Fukuoka-1 (FK), derived from a patient with Gerstmann–Sträussler–Scheinker syndrome, was significantly increased in cultures treated with the macroautophagy inhibitor 3-methyladenine (3MA) but substantially reduced in those treated with the macroautophagy inducer rapamycin. The decrease in FK-derived PrP^Sc^ levels was mediated, at least in part, by the phosphatidylinositol 3-kinase/MEK signalling pathway. By contrast, neither rapamycin nor 3MA had any apparently effect on PrP^Sc^ from either the 22L or the Chandler strain, indicating that the degradation of PrP^Sc^ in host cells might be strain-dependent.

## Introduction

Transmissible spongiform encephalopathies, so-called prion diseases, are fatal neurodegenerative disorders that include Creutzfeldt–Jakob disease in humans, bovine spongiform encephalopathy in cattle and scrapie in sheep. They are transmitted by prions, unconventional infectious agents that mainly consist of proteinase-resistant and β-sheet-rich amyloidogenic isoforms (PrP^Sc^) of the normal host protein PrP (referred to as the conformational isoform, PrP^C^) [1, 2].

The degradation of cellular organelles and cytoplasmic proteins is carried out by a process referred to as autophagy, of which there are three types: macroautophagy, microautophagy and chaperone-mediated autophagy (CMA). In macroautophagy, cytoplasmic proteins or components thereof are incorporated into autophagosome; these vesicles then fuse with lysosomes for subsequent degradation. Macrophagy is the typical cellular degradation pathway and it differs from microautophagy, in which proteins are directly imported into lysosomes, and CMA, in which chaperones recognizing characteristic protein motifs mediate lysosomal transport. Thus, the term autophagy usually indicates macroautophagy [3]. Recently, autophagy was shown to play a crucial role in the clearance of aggregated proteins, such as insoluble amyloid β-proteins, α-synuclein and huntingtin [3–13]. Related studies have focused on the role of the autophagic system in the degradation of PrP^Sc^, with misfolded PrP shown to undergo degradation in the autophagic system in vitro [14] and in vivo by mutated PrP (A116V) transgenic mice [15] and 263K-infected hamsters [16]. In addition, PrP^Sc^ was shown to be remarkably degraded by LiCl-induced autophagy, with different lithium compounds, such as Li_2_CO_3_, LiBr and LiCH_3_COO, strongly provoking autophagy and thus reducing PrP^Sc^ levels in cells infected with mouse strain RML prions [17, 18]. Trehalose, an α-linked disaccharide synthesised by fungi, was shown to induce autophagy and to reduce PrP^Sc^ [19] as well as mutant huntingtin and α-synuclein proteins [20]. Recently, we found that the immune suppressor FK506 (tacrolimus) prevented prion infection in vivo and promoted the degradation of PrP^Sc^ in vitro [21]. The mechanism seems to involve the accelerated clearance of PrP^Sc^ in prion-infected cells via the activation of p62, a cytosolic protein known to mediate both the formation and the degradation of abnormal protein aggregates [22]. Taken together, these observations suggest that PrP^Sc^ produced in the cytoplasm of host cells might undergo autophagic degradation.

In this study, we focused on the cellular signalling cascades related to autophagy and compared the effect of inhibitors/stimulators of this system on PrP^Sc^ degradation, using cell cultures persistently infected with different strains of prions.

## Materials and Methods

### Reagents and Antibodies

Anti-PrP polyclonal antiserum (SS) and M20 (Santa Cruz Biotechnology, Dallas, TX, USA) were mouse and goat polyclonal antibodies, respectively. Anti-PrP SAF61 (SPI-BIO/Cayman Chemical, Ann Arbor, MI, USA), 3F4 (Signet Laboratories, Dedham, MA, USA) and mouse β-actin (Sigma Aldrich, St. Louis, MO, USA) were mouse monoclonal antibodies. Anti-LC3 (Medical & Biological Laboratories, Japan), anti-Beclin-1 and anti- Atg12-Atg5 (both from Cell Signaling Technology, Japan) were rabbit polyclonal antibodies used in the detection of molecules involved in autophagy. The rabbit monoclonal and polyclonal antibodies anti-Lamp1, anti-phosphorylated S6 ribosomal protein (Ser235/236), anti-phosphorylated eIF4B (Ser422), anti-Akt, anti-phosphorylated Akt (Ser473), anti-p44/42 MAPK and anti-phosphorylated p44/42 MAPK (Thr202/204) were purchased from Cell Signaling Technology. Horseradish-peroxidase-conjugated anti-mouse, anti-rabbit (GE Healthcare, Japan) and anti-goat (Jackson ImmunoResearch Laboratories, Baltimore, MD, USA) IgG antibodies were used for western blotting. A detailed description of the procedure has been published [21–23]. 3-methyladenine (3MA), rapamycin, MG132, epoxomicin, LY294002 and PD98059 were purchased from Sigma and Merck Millipore (Merck, Germany). These compounds were dissolved in dimethyl sulfoxide (Sigma) or deionized distilled water. Monodansylcadaverine (Sigma) was used as an indicator of autophagolysosomal complexes and dissolved in ethanol (Nacalai Tesque, Japan).

### Cell cultures

Mouse PrP-overexpressing N2a-58 cells and three lines of these cells persistently infected with prion strains 22L, Fukuoka-1 or Chandler (N2a-22L, N2a-FK or N2a-Ch), were prepared as previously described [23–26]. Briefly, a 10% brain homogenate (BH) was prepared from prion-disease-onset ddY mice and used to infect N2a-58 cells. All animal experiments were approved by the Committees on Animal Care and Use of Nagasaki University and were performed according to their recommendations (Permit No.: 1102170900). The cells were cultured in DMEM (Sigma) containing 10% heat-inactivated foetal bovine serum and 1% penicillin-streptomycin (Life Technologies, Japan) and split every 3 days at a 5 to 10-fold dilution. All cultured cells were maintained at 37°C in 5% CO_2_ in the biohazard prevention area of the author’s institution. In drug treatment studies, cells (2 × 10^4^ cells/well) were grown in 12-well plate for 24 h prior to the addition of each drug. In mouse Atg5 knockdown study, psiRNA-mATG5 plasmid (InvivoGen, USA) was transduced by Fugene 6 (Roche Diagnostics K.K.) as transfectant into N2a-FK cells and the cells were harvested after 48 h. In knockdown study, psiRNA-Luc GL3 plasmid (InvivoGen) as control was used. Starvation conditions were obtained by replacing the growth medium with Hank’s balanced salt solution (HBSS; Wako, Japan) and culturing the cells for 24 h. Fluorescence imaging of autophagic induction was achieved by Lipofectamine LTX (Life Technologies)-mediated transfection of the cells with plasmid pEGFP-LC3, prepared by inserting a mouse LC3 cDNA between the *Bgl*II and *EcoR*I sites in the multiple cloning site (MCS) of pEGFP-C1 (Clontech Laboratories, CA, USA). The plasmid-transfected cells were treated with the various drugs after 24 h and continuously cultured for 24 h. In autophagic level evaluation study using immunoblotting and microscope imaging, to inhibit protein degradation in lysosomes, the cells were pre-treated with 10 mM NH_4_Cl (Nacalai Tesque). After 24 h from NH_4_Cl treatment, the cells were appropriately added drugs to use for each study and cultured for a further 24 h.

### Western blotting

The drug treated cells were harvested with lysis buffer (50 mM Tris-HCl, pH 7.5, containing 150 mM NaCl, 0.5% Triton X-100, 0.5% sodium deoxycholate and 2 mM EDTA). After 2 min of centrifugation at 10,000 × g, the supernatant was collected and its total protein concentration was measured using the BCA protein assay kit (Nacalai Tesque). To detect PrP^Sc^, the protein concentration was adjusted to 5 mg/ml and the samples were digested with 20 μg proteinase K (PK; Sigma)/ml at 37°C for 30 min, followed by boiling for 10 min with sample buffer (50 mM Tris-HCl, pH 6.8, containing 5% glycerol, 1.6% SDS, 100 mM dithiothreitol and a moderate amount of bromophenol blue). After SDS-polyacrylamide (15%) gel electrophoresis, the proteins were transferred onto a PVDF membrane (Immobilon-P; Merck Millipore) which was blocked with 5% skim milk in TBST (10 mM Tris-HCl, pH 7.8, 100 mM NaCl, 0.1% Tween 20) for 1 h at room temperature. The membrane was then reacted with diluted primary (1:1000) and HRP-conjugated secondary antibodies (1:5000). Immunoreactive bands were visualized by ECL prime (GE Healthcare). To quantify the signals, the intensity of each band was measured using the NIH image J software. A detailed description of the methods was previously provided [22].

### Lysosomal purification

Lysosomes were purified from mammalian cells as described previously, with several modifications [27]. All steps were carried out at 4°C unless otherwise noted. Cells at an initial concentration of 1 × 10^9^, corresponding to the number of cells cultured under starvation conditions in four 75-cm^2^ flasks, were grown to 90% confluency and harvested by trypsinization. All subsequent lysosomal isolation steps were performed according to the protocol included in the lysosome isolation kit (Sigma). In brief, the cells were centrifuged at 600 × g for 5 min and resuspended as a 1mL packed cell volume (PCV) in PBS. After the dilution of 2.7 × PCV mL with 1 × extraction buffer, the cells were finally subjected to five freeze-thaw cycles. The degree of breakage was checked until 80–85% of the cells were broken. Cellular debris such as nuclei was removed by centrifugation at 2,000 × g for 10 min and the supernatant further was centrifuged at 20,000 × g for 1 h. The resulting pellet, containing the crude lysosomal fraction (CLF), was resuspended in 1 mL of extraction buffer, as the minimal volume. To enrich the lysosomes, the suspension was further purified by density gradient ultracentrifugation at 150,000 × g for 4 h followed by filling of Diluted Optiprep Fraction (DOF) along with the a part of CLF by 27 to 8% Optiprep Density Gradient Medium solutions, included in the kit, according to the manufacturer’s instructions. After centrifugation, several fractions of the appropriate volume were collected from the top of the gradient. Each fraction was assayed for the amount of Lamp-1, as a standard of lysosomal protein, and for β-actin and PrP^Sc^, which was treated with final 40 μg of proteinase K /ml at 37°C for 30 min.

### Immunocytochemistry

The cells were treated with 10 mM 3MA and 1 μM rapamycin, either separately or together, for 24 h or by incubation in HBSS for 8 h, and then incubated with 0.1 mM MDC in PBS for 30 min at 37°C. The cells were then washed twice with PBS and observed using an Axio Observer Z1 microscope (Carl Zeiss, Deutsch). MDC-positive granules were counted in all treated cells as previously described [21]. For immunofluorescence analysis, the drug-treated cells were fixed for 20 min at room temperature in 4% paraformaldehyde buffer and permeabilized with 0.5% Triton X-100 for 5 min at room temperature. For PrP^Sc^ staining, the cells were treated with 3M guanidine thiocyanate for 5 min, blocked for 1 h at room temperature in TBST containing 5% skim milk and incubated overnight at 4°C with SAF61 antibody (1:100). The cells were washed in PBS and then incubated with Alexa Fluor-488-conjugated secondary antibody (Life Technologies) (1:200) for 60 min at 37°C. In this PrP^Sc^ detection, prion-uninfected N2a-58 cells as a negative control were used. For lysosomal staining, the cells were incubated and pre-stained with 1 μM Lysotracker dye (Life Technologies) for 30 min in a CO_2_ incubator and then fixed as described above. For nuclear staining, the cells were labelled in mounting medium containing the DNA counterstain DAPI. All images were obtained using a confocal laser-scanning microscope 700 (Carl Zeiss). The immunofluorescence staining protocols used in this study were previously described in detail [22].

### Statistical analysis

Student’s t-test and the Mann-Whitney U-test were used in comparisons of two groups, and the one-way ANOVA followed by the Tukey-Kramer test in multiple comparisons. The log-rank test was used to analyse the mortality of prion-infected mice. Statistical analysis of all data was performed using Statcel 2 of the Excel and GraphPad Prism software.

## Results

### PrP^Sc^ degradation is strongly reduced by lysosomal but not by proteasomal inhibitors in N2a-FK cells

**Fig 1:**
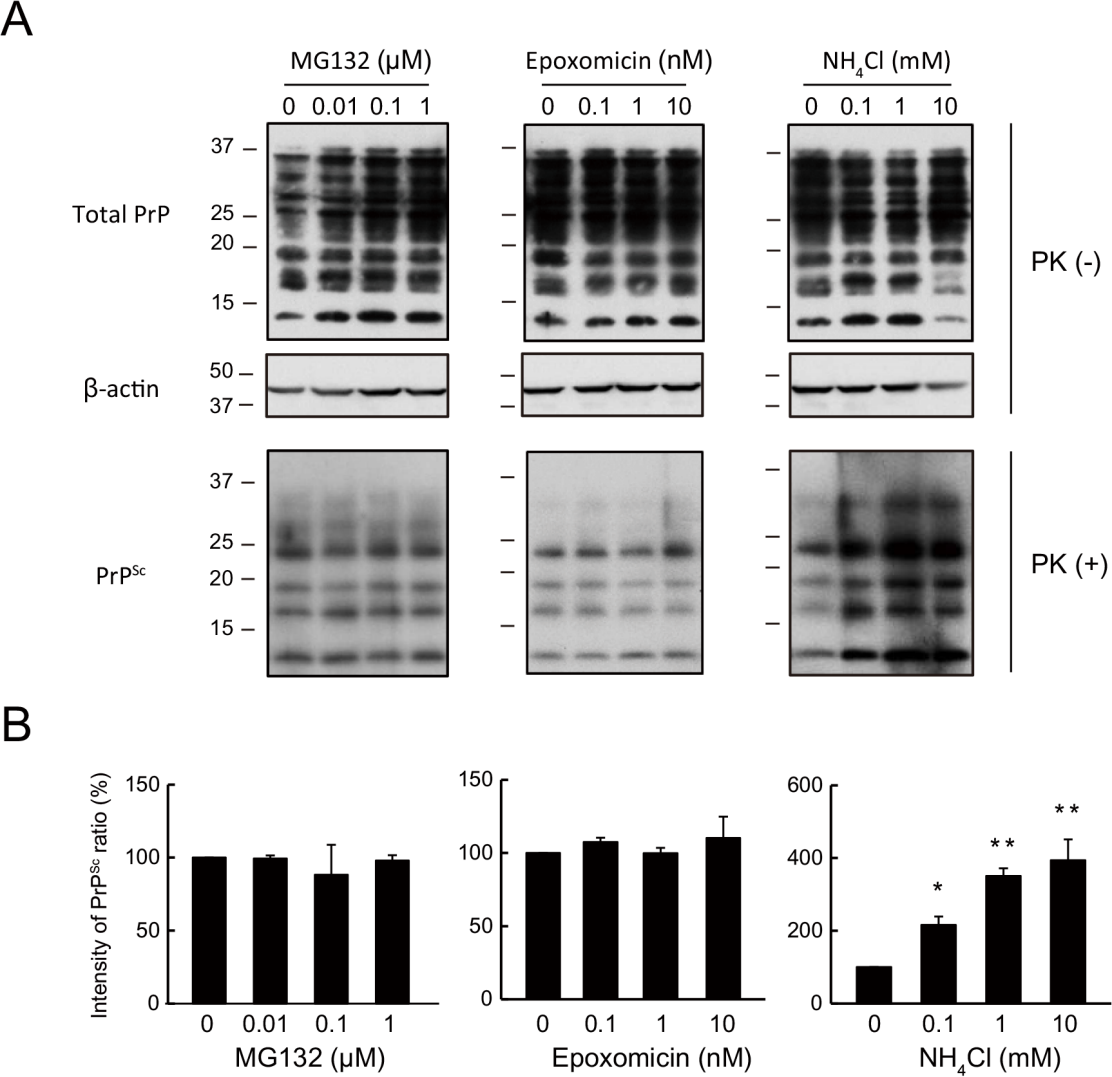
PrP^Sc^ in N2a-FK cells is potently increased by a lysosomal but not by a proteasomal inhibitor. (A) N2aFK cells were treated for 48 h with 0.01 to 1 μM MG132 and 0.1 to 10 nM epoxomicin (Epo) as proteasome inhibitors and 0.1 to 10 mM NH_4_Cl as a lysosomal inhibitor. PK-treated and-untreated N2a-FK cells were loaded at concentrations of 100 and 30 μg protein per lane onto a 15% polyacrylamide gel and subjected to SDS-PAGE. The proteins were detected by western blotting using anti-PrP and -β-actin antibodies. (B) For densitometric analysis, PrP^Sc^ band intensities are expressed as a percentage of those of the negative controls. The results in the graph are the mean ± SD of at least three independent experiments. *p < 0.05 and **p < 0.01 (one-way ANOVA followed by Tukey's test).

It is known that protein degradation system can be largely classified into two groups as ubiquitin-proteasome and lysosome system. Thus, to confirm PrP^Sc^ degradation system in mouse neuroblastoma cells persistently infected with a mouse-adapted prion strain derived from a patient with Gerstmann-Sträussler-Scheinker syndrome, a genetic form of human prion disease (N2a-FK), we investigated about the degradation pathway using proteasomal and lysosomal inhibitors as a familiar pharmacological approach. Treatment of N2a-FK cells with 0.1 to 10 nM epoxomicin or 0.01 to 1 μM MG132, both of which inhibit proteasome activation, had no effect on PrP^Sc^ degradation after 48 h (Fig 1A). However, when the cells were treated with 0.1 to 10 mM NH_4_Cl, PrP^Sc^ degradation was inhibited dose-dependently and significantly (Fig 1A and 1B). These results demonstrate the PrP^Sc^ clearance in N2a-FK cells may relate to the autophagy-lysosomal pathway because it is known that NH_4_Cl will block the late stage of the autophagy-lysosomal pathway in lysosomal system.

### PrP^Sc^ is markedly influenced by inducers and inhibitors of autophagy in N2a-FK persistently prion-infected cells

**Fig 2:**
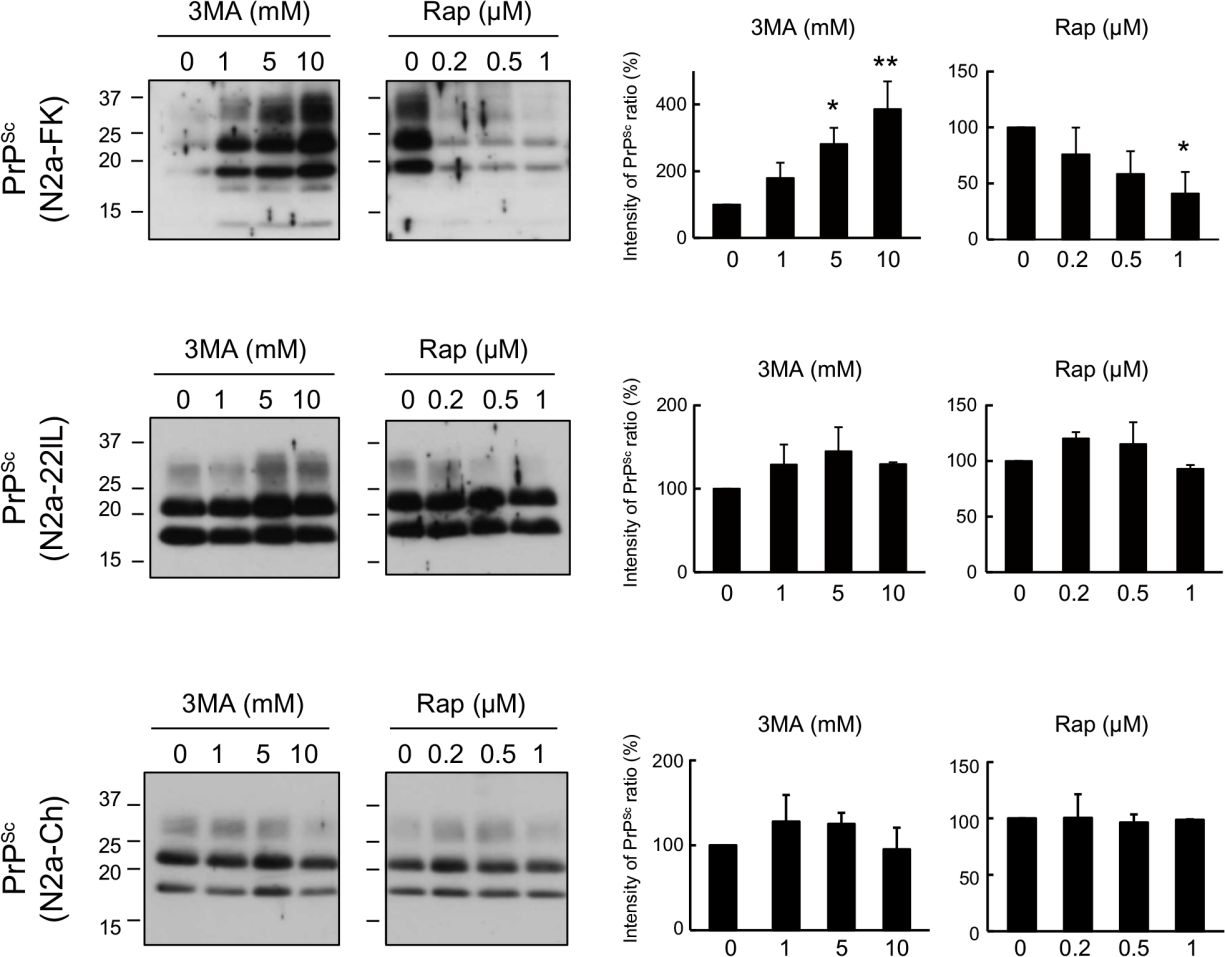
PrP^Sc^ in N2a-FK cells is markedly degraded by the autophagy pathway. Persistently prion-infected cells were treated with 1 to 10 mM of 3-methyladenine (3MA) and 0.2 to 1 μM rapamycin (Rap) for 48 h. Proteinase K (PK)-treated N2a-FK, -22L and-Ch cells, which vary in the prion strains, were loaded at concentrations of 100, 60 and 35 μg protein per lane onto a 15% polyacrylamide gels and subjected to SDS-PAGE. PrP^Sc^ was detected by western blotting using an anti-PrP antibody. For densitometric analysis, the images were scanned and the intensity of each band on the western blotting was quantified with respect to PrP^Sc^ expression levels in drug-treated prion-infected cells, respectively. The results are representative of at least three independent experiments, with each experiment performed in triplicate. *p < 0.05 and **p < 0.01 (one-way ANOVA followed by Tukey's test).

To elucidate the detailed degradation mechanism of PrP^Sc^ in autophagy-lysosomal pathway, we analyzed the effects of rapamycin, a widely used macroautophagy activator that inhibits mTOR, and 3-methyladenine (3MA), a selective inhibitor of macroautophagy that blocks the early stage of the autophagy–lysosomal pathway, by inhibiting type-III phosphatidylinositol 3-kinase (PI3K), on PrP^Sc^ levels in N2a-FK cells. The cells were treated with 1 to 10 mM 3MA and 0.2 to 1 μM rapamycin for 48 h. A dose-dependent increment of PrP^Sc^ was observed in N2a-FK cells treated with 3MA and a dose-dependent reduction in those treated with rapamycin (Fig 2 upper). By contrast, neither drug had any effect on PrP^Sc^ in mouse neuroblastoma cells persistently infected with the scrapie-derived 22L or Chandler strain of prions (N2a-22L and-Ch cells) (Fig 2 middle and bottom).

To determine the level of autophagic activity in N2a-FK cells, we analyzed the expression of the autophagy-related molecules, LC3-II, Beclin-1 and the Atg12-Atg5 complex. The LC3-II / LC3-I ratio was 10- to 40-fold higher in rapamycin-treated cells, but was only slightly increased in 3MA-treated cells. Beclin-1 and Atg12-Atg5 increased in response to rapamycin and decreased in response to 3MA, both in a dose-dependent manner (S1 Fig). Moreover, rapamycin treatment also caused dose-dependent decreases in phosphorylated S6 ribosomal protein and phosphorylated eIF4B protein, two markers of the mTOR pathway (S1 Fig). Furthermore, we also investigated about PrP^Sc^ degradation using Atg5 shRNA and observed the increment of PrP^Sc^ in N2a-FK cells, indicating that the result of other method was also similar to that of chemical compound (S2A Fig).

**Fig 3:**
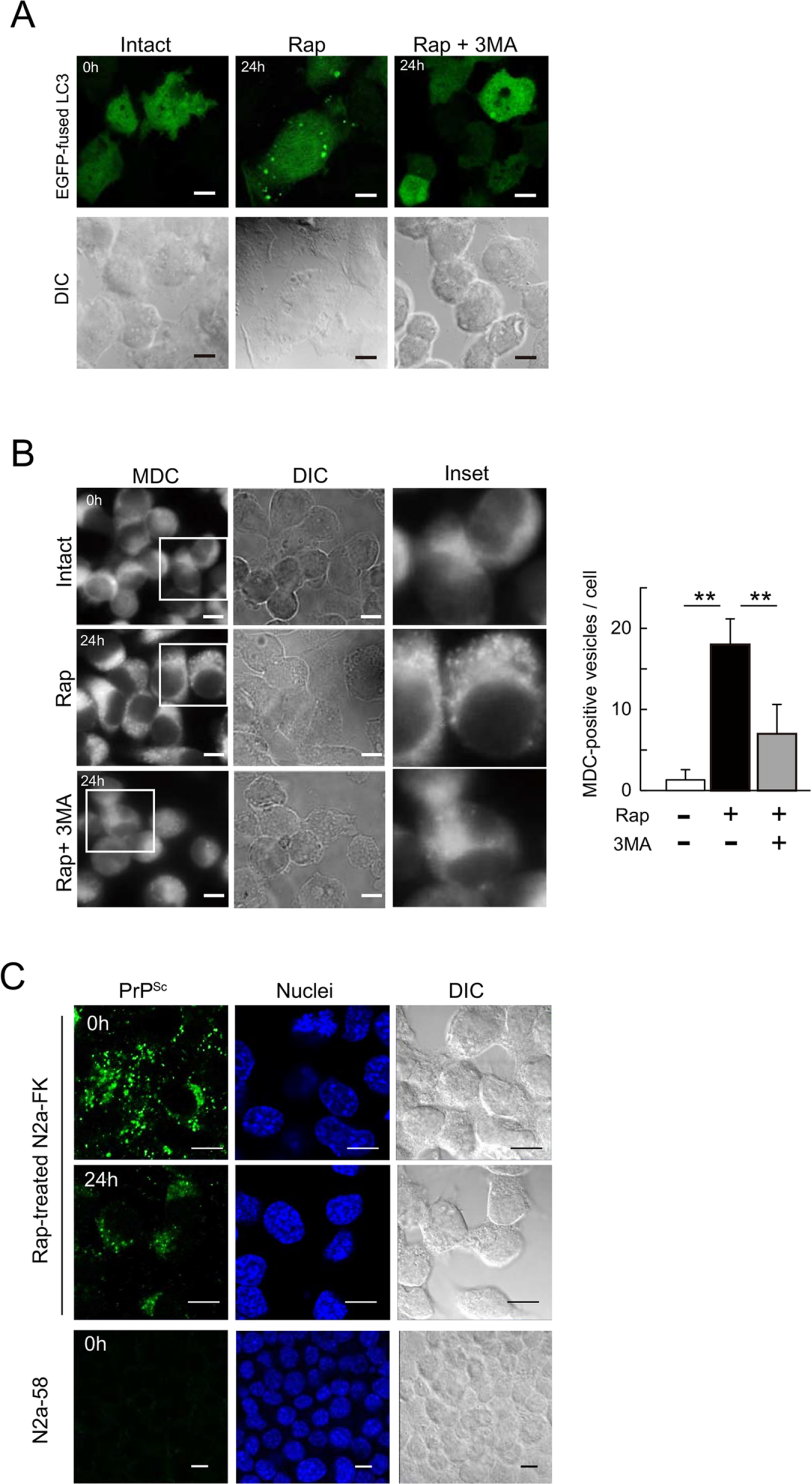
Intracellular formation of autophagolysosomes following rapamycin treatment in N2a-FK cells. (A) To determine autophagic activity in 1 μM rapamycin- or 1 μM rapamycin and 10 mM 3MA-treated N2a-FK cells, autophagosomes were detected in transfectants expressing the EGFP-fused LC3 gene (inserted into plasmid pcDNA 3.1) and the morphological changes in LC3-positive granular vesicles were followed. (B) Autophagolysosomes in cells treated with 1 μM rapamycin for 24 h were visualized using 0.1 mM of monodansylcadaverine (MDC) for 30 min (left, three panels per group). To inhibit rapamycin-induced autophagy, N2a-FK cells were co-treated with 10 mM 3MA. Scale-bars represent 10 μm. To quantify the average number of MDC-labeled autolysosomes in a single cell, the vesicles in the treated cells were shown as a graph represented by the mean ± SD of three independent experiments (right). **p < 0.01 (one-way ANOVA followed by Tukey's test). (C) N2a-FK cells treated with 1 μM rapamycin for 24 h were pre-treated with 3 M guanidine thiocyanate prior to the antibody reaction. PrP^Sc^ in cells was detected using the SAF61 antibody (green) and N2a-58 cells were stained as a negative control. Cell nuclei were counterstained with DAPI (blue). The cells were visualized by confocal laser scanning microscopy. Differential interference contrast (DIC) images were obtained to confirm the consistency of the experimental condition. Scale-bars represent 10 μm.

Accordingly, we examined autolysosome, which is the final structure in the autophagy system, levels by first altering the autophagic vesicles such that they expressed an EGFP-fused LC3 transgene and then staining the autolysosomes with the autofluorescent marker monodansylcadaverine (MDC), which specifically detects autolysosomes [28]. LC3-positive vesicles were increased in N2a-FK cells treated with rapamycin for 24 h, but the increment was suppressed by 3MA (Fig 3A). In previous studies in other mammalian cells, the number of MDC-positive cells in cultures treated with either oridonin or starvation via amino acid deprivation, both of which induce autophagy, was reduced by co-treatment with 3MA [28, 29]. As controls in our study, MDC-labelled vesicles were abundant in N2a-FK cells incubated for 8 h in Hank’s balanced salt solution (HBSS) to induce starvation, whereas the number of vesicles was significantly reduced in starved cells co-treated with 3MA (S3 Fig). Similarly, vesicle induction in rapamycin-treated N2a-FK cells was strikingly reduced when the cells were co-treated with 3MA for 24 h (Fig 3B). Thus, in our system both rapamycin and starvation strongly induced autophagy in persistently prion-infected cells.

The localization of PrP^Sc^ after autophagy induction was determined by immunofluorescence staining of PrP^Sc^ in N2a-FK cells treated with the protein denaturant guanidine [30, 31]. In this method, PrP^C^ in N2a-58 prion-uninfected cells were not detected. After 24 h, the accumulation of PrP^Sc^ in the cytoplasm was reduced compared to 0 h (Fig 3C). Meanwhile, in rapamycin-treated N2a-22L cells high levels of PrP^Sc^ accumulated that strongly localized in the vicinity of nuclei after 24 h (S4 Fig). In HBSS-starved N2a-FK cells, the reduction of PrP^Sc^ was similar to that of rapamycin-treated cells after 24 h (S5 Fig), indicating that PrP^Sc^ might be selectively degraded by the autophagolysosome system in N2a-FK cells.

### The intracellular signalling cascade in the autophagic pathway contributes to PrP^Sc^ clearance in N2a-FK cells

**Fig 4:**
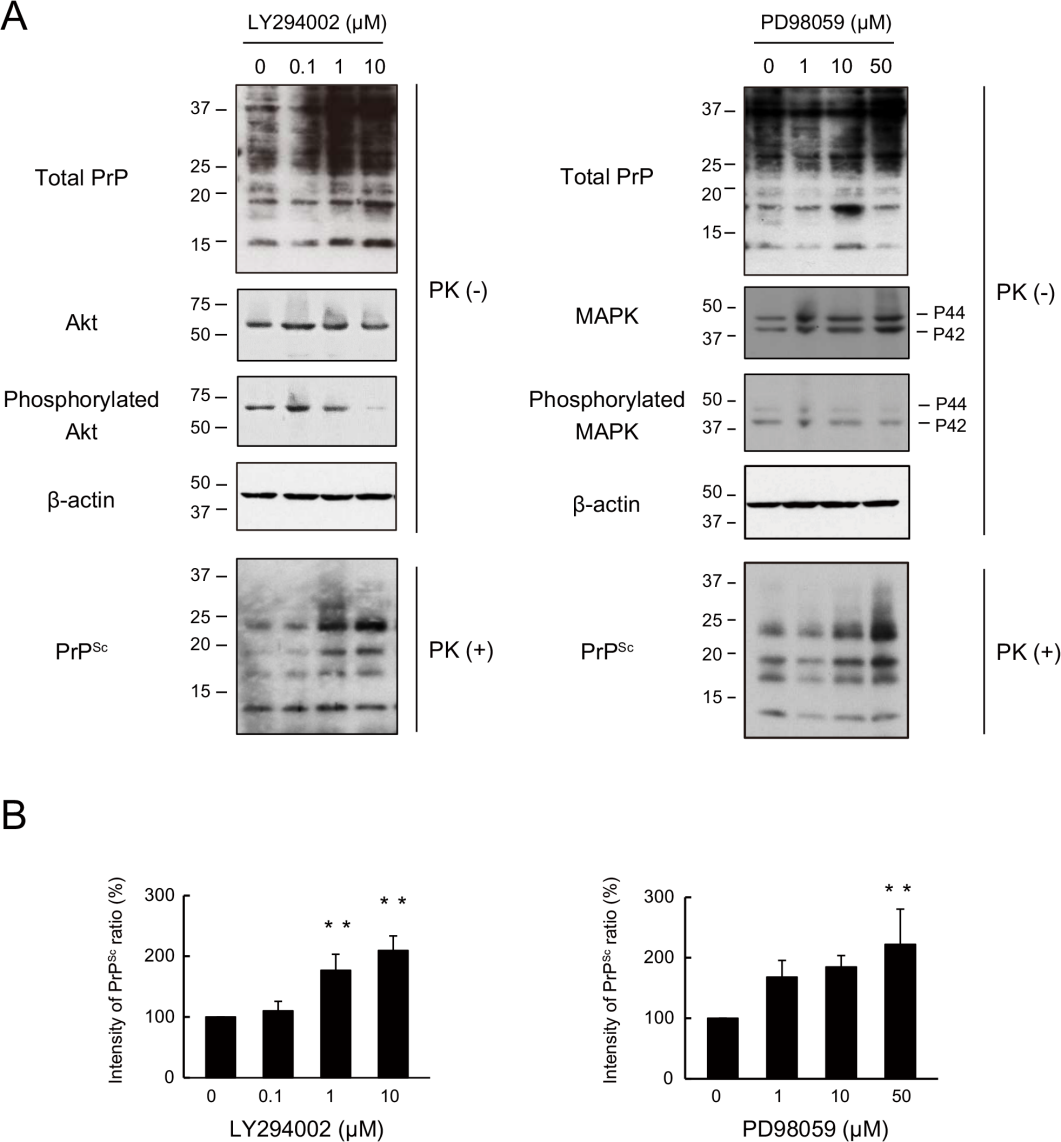
PrP^Sc^ in N2a-FK cells undergoes degradation via upstream intracellular signalling cascades associated with autophagy. (A) N2a-FK cells were treated with 0.1 to 10 μM of the the PI3K inhibitor LY294002 and 1 to 50 μM of the MEK inhibitor of PD98059 for 48 h. PK-treated or-untreated samples were applied at concentrations of 100 and 50 μg protein per lane onto a 15% polyacrylamide gel and subjected to SDS-PAGE. The proteins were analyzed by western blotting using anti-PrP, anti-Akt, anti-phosphorylated Akt (to determine the Akt activation level), anti-p44/p42 MAPK, anti-phosphorylated p44/p42 MAPK (to determine the p44/p42 MAPK activation level) and anti-β-actin antibodies. (B) The effect of these drugs on PrP^Sc^ was determined by quantifying the PrP^Sc^ band intensities as a percentage of those of the negative controls. The results in the graph are the mean ± SD of at least three independent experiments. *p < 0.05 and **p < 0.01 (one-way ANOVA followed by Tukey's test).

**Fig 5:**
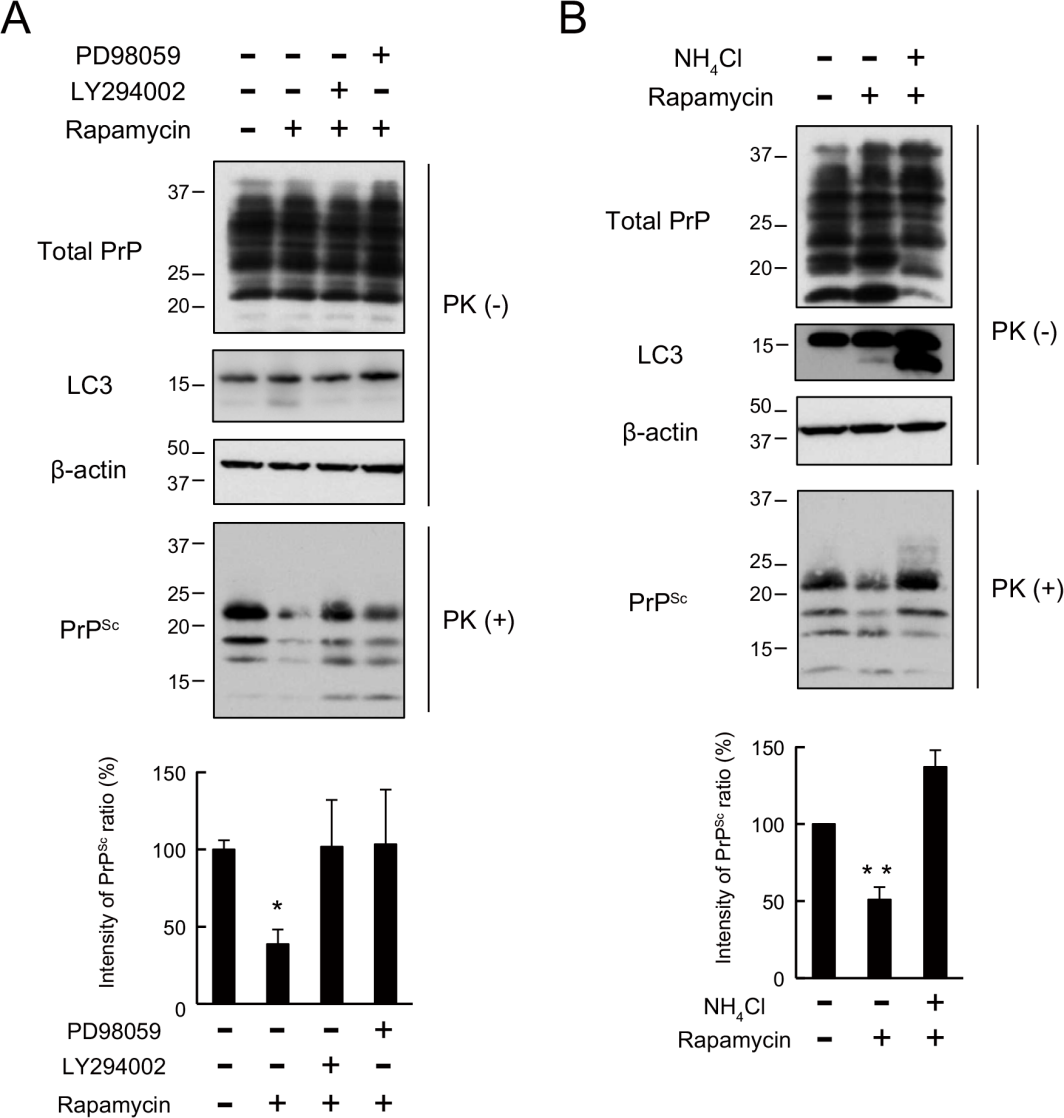
Effects of PI3K, MEK and lysosomal inhibitors on rapamycin-elicited autophagic responses in N2a-FK cells. (A) N2a-FK cells were co-treated with LY294002 (10 μM) or PD98059 (50 μM) along with rapamycin (1 μM) for 48 h. (B) N2a-FK cells were co-treated with NH_4_Cl (10 mM) together with rapamycin (1 μM) for 48 h. Upper: PK-treated or-untreated samples were applied at concentrations of 100 and 50 μg protein per lane onto a 15% polyacrylamide gel and subjected to SDS-PAGE. The proteins were detected by western blotting using anti-PrP,-LC3 and -β-actin antibodies. Bottom: PrP^Sc^ expression levels following drug treatment were quantified in N2a-FK cells. The results are representative of at least three independent experiments, with each experiment performed in triplicate. *p < 0.05 and **p < 0.01 (one-way ANOVA followed by Tukey's test).

Next, to investigate whether the intracellular signalling cascade of the autophagic pathway had an effect on the degradation of PrP^Sc^, we tested the effects of PI3K inhibitor, LY294002. The pharmacological properties of LY294002 include an ability to inhibit the macroautophagic degradation of proteins in mammalian cells at autophagic sequestration step [32, 33] and to block PI3K-dependent Akt phosphorylation and kinase activity [34, 35]. The effect of LY294002 as a PI3K inhibitor was confirmed by the dose-dependent decrease in phosphorylated Akt levels in N2a-FK cells after 48 h (Fig 4A). In similarly treated cells, PrP^Sc^ levels significantly and dose-dependently increased (Fig 4B), confirming the role of PI3K in PrP^Sc^ degradation. Next, to investigate the relationship between PrP^Sc^ degradation and the intracellular signalling cascade downstream of PI3K, we assessed the effects of PD98059, an inhibitor of mitogen-activated protein kinase/extracellular signal-regulated kinase (MAPK/ERK: MEK), on PrP^Sc^ degradation. MEK signalling regulates autophagy by regulating Beclin-1, through the mTOR pathway [36, 37]. PD98059 inhibits MEK signalling such that the phosphorylation of downstream ERK 1/2 (ERK1/2: P44/P42) is prevented. A role for MEK signalling was verified by the decrease in phosphorylated MAPK levels in N2a-FK cells (Fig 4A). When these cells were treated with 50 μM of the inhibitor for 48 h, PrP^Sc^ levels were significantly increased (Fig 4B). Subsequently, we asked whether PrP^Sc^ degradation by rapamycin-induced autophagy was blocked by these inhibitors of autophagy-related signalling cascades (Fig 5). Indeed, in N2a-FK cells the rapamycin-induced decrease in PrP^Sc^ was significantly overcome by co-treatment with either LY294002 (10 μM) or PD98059 (50 μM) for 48 h (Fig 5A). Moreover, the reduction in PrP^Sc^ was significantly recovered in cells co-incubated for 48 h with rapamycin and the lysosomal inhibitor NH_4_Cl (Fig 5B). These results provide evidence of the involvement of PI3K and MEK signalling in autophagy-mediated degradation of PrP^Sc^ in N2a-FK cells.

### Nearly all of the PrP^Sc^ in N2a-FK cells is degraded in lysosomes

**Fig 6:**
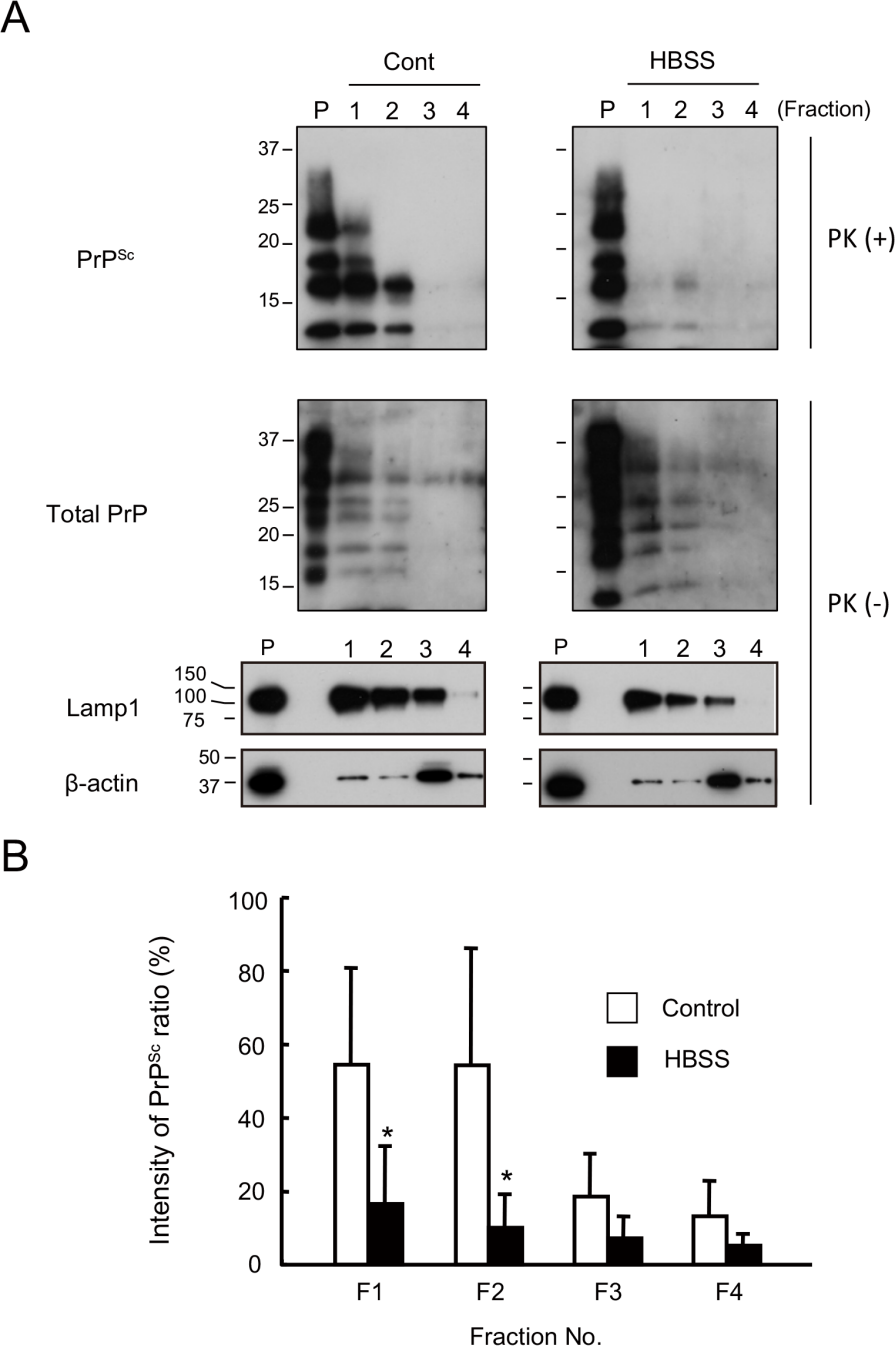
PrP^Sc^ levels in the lysosomal fraction are reduced following autophagy in starved cells. (A) Lysosomes were isolated by the fractionation of N2a-FK cells incubated or not in HBSS for 8 h, as an inducer of autophagy. PK-treated or-untreated gradient fractions were applied at one-tenth of their original volume per lane onto a 15% polyacrylamide gel and subjected to SDS-PAGE. The proteins were detected by western blotting using anti-PrP, anti-Lamp1 (as a lysosomal marker) and anti-β-actin (as the internal standard) antibodies. Fractions 1 to 3 contained high-quality, purified native lysosomes. Positive controls consisted of cell lysates containing 15 μg of protein before purification (P). (B) To quantify ratio of the PrP^Sc^ degradation in the isolated lysosomes, PrP^Sc^ band intensities were measured as a percentage of those of the controls. Empty columns indicate the control, non-treated fractions and shaded columns the HBSS-treated fractions. The results in the graph are the mean ± SD of at least three independent experiments. *p < 0.05 (Student's t-test).

Autophagolysosomes are the product of autophagosome and lysosome fusion and their formation is a necessary step in protein clearance by the autophagy system. In HBSS-starved N2a-FK cells, the accumulation of PrP^Sc^ was co-localized in lysosomes in the vicinity of nuclei after 8 h, and the PrP^Sc^ was reduced after 24 h like rapamycin treatment (Fig 3C and S5 Fig). To confirm the localization of PrP^Sc^ within the lysosomal fraction after autophagic induction, we followed the subcellular localization of PrP^Sc^ and the lysosomal marker Lamp1 in N2a-FK cells by isolating a pure lysosomal fraction. Under control conditions, PrP^Sc^ localized to the same lysosome-rich fractions as Lamp1 (Fig 6A, left, lanes 1 to 3). However, in N2a-FK cells in which autophagy was induced by starvation conditions (8 h of HBSS treatment), although total PrP, Lamp1 and β-actin band intensities were similar, there was little PrP^Sc^ in the Lamp1 fractions (Fig 6A, right, lanes 1 to 3). Quantification of the ratio of PrP^Sc^ in each fraction (F1 to F4), based on band intensity, showed significantly less PrP^Sc^ in the lysosome-rich fractions (F1 and F2) of HBSS-starved cells treatment than in those of control cells (Fig 6B), suggesting that under normal conditions PrP^Sc^ might be almost completely degraded in the lysosomal fraction, via the autophagic system.

## Discussion

In this study, PrP^Sc^ in N2a-FK cells was degraded by an autophagic process enhanced by the inducer rapamycin. By contrast, rapamycin had no effect on PrP^C^ in N2a-58 cells (S2B Fig), indicating that autophagy might be one of the degradation system involved in PrP^Sc^ but not in PrP^C^ degradation. Recently, Xu Y, et al. reported that scrapie-derived PrP^Sc^ is degraded by mTOR-related autophagic system via FBXW7 protein [38]. It is possible that GSS-derived PrP^Sc^ may also relate to the degradation system mediating FBXW7, but the elucidation of its option will need to be further investigated.

The site-specific proteolysis of LC3-I (18 kDa) to LC3-II (16 kDa) is indicative of autophagic activity, as are Beclin-1 and the Atg12-Atg5 complex. The latter are required for nucleation of the phagophore and maturation of the autolysosome in mediating autophagosome formation. In our study, LC3 activation in rapamycin-treated N2a-FK cells was approximately 40-fold stronger than in non-treated control cells (S1 Fig). Rapamycin also increased the levels of Beclin-1 and the Atg12-Atg5 complex, both of which were reduced by 3MA treatment, and decreased markers of the mTOR signalling as phosphorylated S6 ribosomal protein and phosphorylated eIF4B protein (S1 Fig). It already have been confirmed that expression of Beclin1 and Atg5 were increased by starvation-induced activation of cardiac autophagy in mice and that of neonatal mice to supply the amino acids for energy homeostasis [39, 40]. In N2a-FK cells, some autophagy-related proteins also may be produced by the same mechanism after autophagic activation. However, the detailed mechanism remains to be determined. These results indicate that PrP^Sc^ in N2a-FK cells might be degraded by the canonical autophagic system, which is induced by rapamycin.

In autophagic signalling, LY294002 specifically blocks PI3K-dependent Akt phosphorylation and kinase activity, resulting in a drastic inhibition of autophagy [32–35]. In another study, the activation of autophagy by HBSS was more strongly prevented by LY294002 than by wortmannin, another inhibitor of PI3K [37]. Here we showed that PrP^Sc^ levels in N2a-FK cells are regulated by an autophagic system involving PI3K and MEK signalling (Figs 4 and 5). In Beclin-1-dependent autophagic activation, an important pathway is that in which Bcl2 is phosphorylated by phosphorylated ERK. In HBSS-starved cells, phosphorylated ERK is suppressed by PD98059 via the inhibition of P44/P42 MAPK, and autophagic sequestration is prevented by increment of Beclin-1 and unphosphorylated Bcl2 complex [37]. In autophagic sequestration inhibitory system by nitric oxide (NO), disruption of hVps34/Beclin-1 complex formation is leaded by increment of Beclin-1/Bcl2 interaction after phosphorylated Bcl2 reduction due to inhibition of JNK1 by NO [41]. It is furthermore likely that PD98059 inhibits autophagy via MAPK-regulated mTOR signalling [42]. Thus, the mechanism underlying the autophagic degradation of PrP^Sc^ in N2a-FK seems to involve a pathway that includes mTOR, PI3K and MEK signalling.

In this study, we were not able to find the evidence of the autophagic degradation of PrP^Sc^ in N2a-Ch and N2a-22L cells, whereas it has reported that PrP^Sc^ in RML-inefcted N2a cells significantly reduced after rapamycin treatment [17, 19]. Likewise, we previously reported that PrP^Sc^ in N2a-FK cells was degraded by autophagy in a process activated by FK506 (tacrolimus) [21], while Karapetyan et al. showed that tacrolimus reduces PrP^Sc^ in RML-infected cells in the absence of autophagic activation [43]. According to Kawasaki et al., compound-B strongly reduces PrP^Sc^ of the RML strain but had only marginally effects on prion strains 22L and FK-1 [44]. These results raise the question, why does the degradation of PrP^Sc^ differ for each prion strain? A difference in the biochemical properties of PrP^Sc^ from different strains such that they differ in their sensitivity to proteolytic enzymes is unlikely because there is no difference in the conformational stabilities of PrP^Sc^ in N2a-22L and-FK cells following their denaturation by guanidine hydrochloride [45]. It was previously reported that low-dose and long-term treatment of 22L- and RML-prion infected GT-1 cells with rapamycin reduces PrP^Sc^ levels, by suppressing protein translation [46]. However, similar experiments were beyond the scope of this study. Evidence of alternative pathways of PrP^Sc^ degradation comes from a study showing that both tamoxifen and 4-hydroxytamoxifen decrease PrP^Sc^ in an autophagy-independent manner, by bringing PrP to lysosomes [47], indicating that the degradation mechanism of PrP^Sc^ might have various pathway. Thus, these verification need to be further examined using various prion strains.

In summary, our results demonstrated that PrP^Sc^ in Fukuoka-1 prion strain-derived cells might be efficiently degraded in canonical autophagic system-dependently compared with those in other prion strains-derived cells. This finding indicates that we might have to alter the therapeutic strategies by patients with Creutzfeldt-Jakob disease, and suggests the need for new therapeutic strategies, such as the use of autophagy-inducing compounds, in patients with Gerstmann-Sträussler-Scheinker syndrome. Further studies of the autophagy-mediated degradation of PrP^Sc^ will provide additional therapeutic insights and the necessary advances to one day allow the complete cure of prion diseases.

## Supporting Information

S1 Fig The effect of autophagy-related proteins in N2a-FK cells after 3MA and rapamycin treatment. N2a-FK cells were treated with 1 to 10 mM of 3-methyladenine (3MA) and 0.2 to 1 μM rapamycin (Rap) for 48 h. PK-untreated samples containing 10 to 30 μg of protein were used to investigate the expression of total PrP, LC3, Beclin-1 and the Atg12-Atg5 complex as indicators of autophagic flux. To confirm the levels of autophagy, LC3-II/-I ratio was assessed by LC3 blotting. β-actin was used as the internal standard for each sample. Phosphorylated S6 ribosomal protein (Ser235 and Ser236) (p-S6) and phosphorylated eIF4B protein (Ser422) (p-eIF4B) levels, as indicators of a rapamycin effect, were performed with western blotting.
(EPS)

S2 Fig The effect of mAtg5 shRNA in prion-infected cells and that of 3MA and rapamycin in prion–uninfected cells. (A) N2a-FK cells which were transduced with mouse Atg5 (mATG5) or Luc-GL3 shRNA plasmids were harvested after 48 h. The psiRNA Luc-GL3 plasmid was used as control. The PrP^Sc^, Atg12-Atg5 complex and β-actin were detected by western blotting and PrP^Sc^ levels were quantified in the cells. (B) PK-untreated samples in N2a-58 prion-uninfected cells which treated with 3MA and Rap for 48 h were used 40 μg protein per lane, and investigated PrP^C^ expression in drug-treated cells using immunoblotting. PrP^C^ levels were quantified in the cells. The results are representative of at least three independent experiments, with this experiment performed in triplicate.
(EPS)

S3 Fig MDC staining for autophagolysosome detection following HBSS treatment in N2a-FK cells. Autophagolysosomes in cells treated with HBSS for 8 h were visualized using 0.1 mM of monodansylcadaverine (MDC) for 30 min (left, three panels per group). Right panels show inset images. To inhibit HBSS-induced autophagy, N2a-FK cells were co-treated with 10 mM 3MA. Scale-bars represent 10 μm. To quantify the average number of MDC-labeled autolysosomes in a single cell, the vesicles in the treated cells were shown as a graph represented by the mean ± SD of three independent experiments (right). **p < 0.01 (one-way ANOVA followed by Tukey's test).
(EPS)

S4 Fig PrP^Sc^ in the N2a-22L cells were not reduced by autophagy triggering rapamycin treatment. The rapamycin-treated the N2a-22L cells were pre-treated with 3M guanidine thiocyanate, prior to antibody reaction. PrP^Sc^ was detected by SAF61 antibody (green). Cell nuclei were counterstained with DAPI (blue). All images were visualized by CLSM700. The differential interference contrast (DIC) images were demonstrated to confirm the consistency in all experimental condition. Scale-bars represent 10 μm.
(EPS)

S5 Fig Intracellular localization of PrP^Sc^ following HBSS-induced autophagy in prion-infected cells. PrP^Sc^ was analyzed in N2a-FK cells treated with HBSS. The cells were visualized by confocal laser scanning microscopy. Differential interference contrast (DIC) images were obtained to confirm the consistency of the experimental condition. Scale-bars represent 10 μm.
(EPS)
